# Dental Caries on Deciduous Molars among Children Visiting Dental Outpatient Department of a Tertiary Care Centre

**DOI:** 10.31729/jnma.8360

**Published:** 2023-12-31

**Authors:** Bhoj Raj Adhikari, Mamata Shakya, Nisha Bhatta, Sumita Upadhya, Swagat Kumar Mahanta

**Affiliations:** 1 Department of Oral Medicine and Maxillofacial Pathology, Kathmandu University School of Medical Sciences, Dhulikhel, Kavre, Nepal; 2 Department of Oral and Maxillofacial Surgery, Kathmandu University School of Medical Sciences, Dhulikhel, Kavre, Nepal; 3 Department of Pediatric and Preventive Dentistry, Kathmandu University School of Medical Sciences, Dhulikhel, Kavre, Nepal; 4 Department of Community and Public Health Dentistry, Kathmandu University School of Medical Sciences, Dhulikhel, Kavre, Nepal

**Keywords:** *children*, *dental caries*, *molars*, *prevalence*

## Abstract

**Introduction:**

Dental caries is one of the most common, yet preventable childhood diseases. The aetiology of dental caries lies in the interplay between host, microorganism, substrate, and time. Risk factors for dental caries include physical, biological, environmental, behavioural, and lifestyle-related factors such as high numbers of cariogenic bacteria, inadequate salivary flow, insufficient fluoride exposure, poor oral hygiene, inappropriate methods of feeding infants, and poverty.

**Methods:**

This descriptive cross-sectional study was conducted on children who visited the dental outpatient department of Kathmandu University School of Medical Sciences, Dhulikhel Hospital from 15 June 2023 to 30 July 2023. Ethical approval was taken from the Institutional Review Committee of the same institute. A convenience sampling method was used. The point estimate was calculated at a 95% Confidence Interval.

**Results:**

The prevalence of dental caries on deciduous molars in children was 252 (93.33%)(90.35-96.30, 95% Confidence Interval). One-third of the children had visited dental clinics for the first time. Lack of topical fluoride application was seen in the majority of the children.

**Conclusions:**

The prevalence of dental caries on deciduous molars among pediatric patients was found to be higher than in other studies done in similar settings.

## INTRODUCTION

Dental caries is one of the most common, yet preventable childhood diseases.^1^ The aetiology of dental caries lies in the interplay between host, microorganism, substrate and time. Risk factors for dental caries include lifestyle-related factors among others.^1,2^ The socioeconomic status of the family can influence parents' perceptions regarding the oral health of their children. A tooth loss in a child at an early age affects the function, esthetics and eruption pattern of permanent teeth as well. Neither all teeth nor all surfaces in each tooth are equally susceptible to dental caries.^3^ Among the teeth on deciduous dentition, molars are most commonly affected by dental caries.^4,5^

Although studies on dental caries have been done extensively, to the best of our knowledge, deciduous molars have never been taken into concern in Nepal.

This study aimed to find the prevalence of dental caries in deciduous molars in children presenting to the dental outpatient department.

## METHODS

This descriptive cross-sectional study was conducted on children who visited the dental department of Kathmandu University School of Medical Sciences Dhulikhel Hospital (KUSMS-DH), Dhulikhel, Kavre, Nepal from 15 June 2023 to 30 July 2023. Ethical approval was obtained from the Institutional Review Committee of the same institute (IRC-KUSMS Approval No: 69/23) and informed consent was taken from the parents of all the study participants prior to the study. Informants of children who gave informed consent were included in this study. The age of the children in the present study were between 2 years and 14 years. The exclusion criteria were the children below two years of age whose deciduous molars had not erupted and those in whom the deciduous molars were replaced by permanent premolars. A convenience sampling method was used.

The sample size was calculated using the following formula:


$$\begin{array}{ll} \text{n} & ={\text{Z}}^{2}\times \frac{\text{p}\times \text{q}}{{\text{e}}^{\text{2}}}\\ & =1.96^{2}\times \frac{0.50\times 0.50}{0.06^{2}}\\ & =267 \end{array}$$

Where,

n = minimum required sample size
Z = 1.96 at 95% Confidence Interval (CI)
p = prevalence taken as 50% for maximum sample size calculation
q = 1-p
e = margin of error, 6%

The minimum sample size was 267, however, 270 children were included.

After obtaining informed and written consent from the parents, the demographic details of the patients were recorded. A brief history was taken which included children's previous dental exposure, professional topical fluoride application and parents' educational status. Parents' educational level was categorized based on completion of formal school education (SLC/SEE or equivalent). Among both the parents, the one with a higher level of education was taken into consideration. Clinical examination of all the deciduous molars was done using standard diagnostic tools including the mouth mirror, probe and explorer. Adjuvant aids including the use of dental floss and radiographs were employed wherever necessary. The deciduous molars were examined, and then, decayed (d), indicated for extraction/missing due to dental caries (e) and filled (f) were identified and recorded using visual inspection, probing and interproximal flossing.^6^

Data were entered in Microsoft Excel and and analyzed using IBM SPSS Statistics version 23.0. Point estimate and 95% CI were calculated.

## RESULTS

Among 270 children, dental caries was seen in 252 (93.33%) (90.35-96.30, 95% CI). Among them, the male and female children were 150 (59.52%) and 102 (40.48%) respectively (Table 1).

**Table 1:** Summary of the clinical findings (n= 252).

Sex	n (%)
Male	150 (59.52)
Female	102 (40.48)
**Previous dental exposure**	
First dental visit	82 (32.54)
Previously visited	170 (67.46)
Professional topical fluoride application	20 (7.94)
**Educational status of parents**	
Below completion of the school	90 (35.71)
Above school level	162 (64.29)

In each individual, the number of infected molars was in the range of one to eight with 22 (8.73%) children having all the deciduous molars infected. The mean dental caries in each individual was found to be 4.16±2.10 teeth. Of all the participants, tooth 84 was found to be most infected counting to 128 (50.79%) (Table 2).

**Table 2:** DMFT findings of all the teeth in the participants (n= 252).

Teeth Number	Decayed n (%)	Missing n (%)	Filled n (%)
54	134 (53.17)	-	2 (0.79)
55	154 (61.11)	6 (2.38)	8 (3.17)
64	134 (53.17)	4 (1.58)	11 (4.36)
65	144 (57.14)	4 (1.58)	7 (2.77)
74	144 (57.14)	4 (1.58)	4 (1.58)
75	136 (53.96)	4 (1.58)	4 (1.58)
84	128 (50.79)	1 (0.39)	8 (3.17)
85	154 (61.11)	-	8 (3.17)

Among the 252 children, 82 (32.54%) visited the dental clinic for the first time, while the remaining 170 (67.46%) had previous dental exposure and received some form of dental treatment. Only a small portion of patients 20 (7.94%) previously had the professional topical fluoride application while the remaining 232 (92.06%) had not received the same.

The highest education level of any one of the parents was found to be below completion of formal school in 90 (35.71%) children and 162 (64.29%) children had their parents completed the school level of education.

## DISCUSSION

Dental caries in deciduous teeth is a common chief complaint for which a pediatric patient is usually brought to the dental clinic. Dental caries being an infectious disease may cause pain, abscess, and swelling and may even complicate cellulitis in children. The present study showed a prevalence of dental caries in the deciduous molars in children to be 93.33%. Among all the teeth on deciduous dentition, molars are most commonly affected by dental caries.^3-5^ The increased rate of dental caries in these teeth is mainly attributed to their morphological features (presence of deep pits and fissures on their occlusal surface) and longer duration of functional status in the oral cavity. Previous studies have shown a wide range of differences in the prevalence of dental caries.^5,8^ A study conducted in the age range very close to the present one has shown a prevalence of 80.6% while studying overall dentition.^6^ In another study conducted on school-going children, the prevalence was found to be between 64 to 78% at different age groups in the overall dentition.^9^ Anterior teeth in the human jaw are less prone to dental caries, as compared to molars, because of their self-cleansing property attributed to their morphological features and location in the oral cavity. This might be one of the reasons for the higher prevalence of dental caries when only molars are studied in our study in contrast to overall dentition in other studies. Another factor responsible for the higher prevalence of dental caries in the present study was the lack of professional topical fluoride application in the teeth that we studied. Although the majority of the participants had visited a dentist, a very small number of them were exposed to topical fluoride application on teeth. Professional topical fluoride applied on the surface of the teeth reacts with the hydroxyapatite crystals on the surface of enamel and forms the fluorapatite crystals making the teeth resistant to dental caries. A total of 92.06% of the patients participating in the present study have never applied topical fluoride which might be another reason for the teeth to be highly prone to decay.

Children are in the critical age group and depend on guardians for proper supervision. Previous studies have shown a higher incidence of dental caries in children itself, as compared to adults and adolescents.^4,5,9^ Dental caries is a multifactorial infectious disease, a lot of factors on a child's behaviour also affect it.^1^ Based on previous studies, a prevalence of 61.8% was noticed in low socioeconomic groups as compared to 49.1% in high socioeconomic status people.^9^ Previous studies have shown that children from socio-economically vulnerable families with lower parental educational levels have a higher prevalence rate of dental caries.^10^ In the present study, only 64.29% of the children had at least one of their parents completed a minimum school level of education. In our study mean dental caries was found to be higher in children whose parent's highest education level was below schooling. Their food habits, oral hygiene habits, deleterious habits, dental exposures and, knowledge and attitudes of their parents might be the determining factor in having higher dental caries in our study population. Dental caries is the primary cause of oral pain and tooth loss.^9^ Children having dental caries in their primary teeth were three times more likely to develop caries in their permanent teeth.^11^ A tooth loss in a child at an early age affects the function, esthetics and eruption pattern of permanent teeth as well.

The present study has a few limitations. The present study examined children, representing a wide age range and the study was conducted in the university hospital. The children of this age are school-going children, who go to school and may not be available during the time of day of the examination for the research purpose. Clinical examination with extended time might be helpful to incorporate additional study.

## CONCLUSIONS

The prevalence of dental caries on deciduous molars among children was found to be higher than in other studies done in similar settings. The early preventive and curative measures of dental caries in children will help to improve oral health.
